# Graphene Aerogels for In Situ Synthesis of Conductive Poly(para-phenylenediamine) Polymers, and Their Sensor Application

**DOI:** 10.3390/mi11070626

**Published:** 2020-06-27

**Authors:** Sahin Demirci, Mehmet Can, Nurettin Sahiner

**Affiliations:** 1 Department of Chemistry & Nanoscience and Technology Research and Application Center, Canakkale Onsekiz Mart University Terzioglu Campus, Canakkale 17100, Turkey; sahindemirci@gmail.com (S.D.); mehmetcann20@gmail.com (M.C.); 2 Department of Chemical and Biomolecular Engineering, University of South Florida, Tampa, FL 33620, USA; 3 Department of Ophthalmology, Morsani College of Medicine, University of South Florida, 12901 Bruce B. Downs Blvd, MDC21, Tampa, FL 33612, USA

**Keywords:** graphene oxide, graphene aerogel, poly(para-phenylenediamine), GA/conductive polymer composite, conductivity, sensor

## Abstract

In this study, macroporous graphene aerogels (GAs) were synthesized by chemical reduction of graphene oxide sheets and were used as a support material for in situ synthesis of conductive poly(para-phenylenediamine) (p(p-PDA)). The in situ synthesis of p(p-PDA) in GA was carried out by using a simple oxidation polymerization technique. Moreover, the prepared conductive p(p-PDA) polymers in the networks of GAs were doped with various types of acids such as hydrochloric acid (HCl), nitric acid (HNO_3_), sulfuric acid (H_2_SO_4_), phosphoric acid (H_3_PO_4_), respectively. The prepared GA and different acid-doped forms as GA/p(p-PDA) composites were characterized by FT-IR, TGA, and conductivity measurements. The observed FT-IR peaks at 1574 cm^−1^, and 1491 cm^−1^, for stretching deformations of quinone and benzene, respectively, confirmed the in situ synthesis of P(p-PDA) polymers within GAs. The conductivity of GAs with 2.17 × 10^−4^ ± 3.15 × 10^−5^ S·cm^−1^ has experienced an approximately 250-fold increase to 5.16 × 10^−2^ ± 2.72 × 10^−3^ S·cm^−1^ after in situ synthesis of p(p-PDA) polymers and with HCl doping. Conductivity values for different types of acid-doped GA/p(p-PDA) composites were compared with the bare p(p-PDA) and their undoped forms. Moreover, the changes in the conductivity of GA and GA/p(p-PDA) composites upon CO_2_ gas exposure were compared and their sensory potential in terms of response and sensitivity, along with reusability in CO_2_ detection, were evaluated.

## 1. Introduction

Over the past decades, carbon-based materials have been the focus of eminent interest in both scientific and industrial fields due to their captivating mechanical, electrical, thermal, and physicochemical properties, as well as their environmentally friendly nature and economically viable accessibility that has collectively led to the emergence of diverse structures including carbon nanotubes (CNTs), carbon dots, mesoporous carbon, and graphene and so on [1,2,3,4]. Particularly, amongst all these materials, graphene has come into great prominence owing to its distinct characteristics such as great thermal and electrical conductivity, high stiffness, elasticity, and unique hexagonal monoatomic lattice structure with the high theoretical surface area of ~2630 m^2^/g in the pristine form [5,6,7]. Graphene-based materials have been widely implemented in various areas [6,7,8] including solar cells [9,10,11], anti-corrosion coatings [12], electrode materials [13,14], sensors [14,15], biosensors [16,17,18], biomedical devices [2,19,20,21], and microelectronics [22], e.g., light-emitting diodes [23,24], displays [25,26,27], batteries [28,29] and supercapacitors [30,31]. However, innate functionalities of pristine graphene are only partially inherited into their incorporated polymer composites, mainly because of isolation and aggregation tendencies of graphene sheets, and its chemical stability most likely due to strong π-π interactions [5,32]. Chemically modified graphene forms such as graphene oxides (GOs) have been produced by decorating 2D graphene sheets with various oxygen-containing moieties such as epoxy, hydroxyl, and carboxylic acid groups to surpass aggregation behaviors of graphene sheets [7,33]. Several methods of GO preparation such as Hummers, Staudenmaier, Hoffmann, and Brodie methods and some of their modified forms are the most established ones [7]. GOs possess a good aqueous dispersibility and are electrical insulators because of the spoilage of the sp^2^ hybridization. Preparation of reduced GOs (r-GOs) can be achieved to solve poor conductivity of GOs by treating them with different reducing agents such as hydroquinone, diazene (N_2_H_2_), hydrazine(N_2_H_4_), sodium borohydride(NaBH_4_), sodium hydroxide (NaOH), sodium sulfide (Na_2_S), sodium bisulfate (NaHSO_3_), ascorbic acid (C_6_H_8_O_6_), and hydroiodic acid (HI) [34,35]. GOs have been reported in preparation for a variety of composite materials [36] such as metal composites [37], various polymers, hydrogels [38], and cryogels [39].

Aerogels are a specific type of 3D network with ultra-low-density and extreme surface areas, highly porous structure, and great mechanical elasticity, and have been exploited in numerous applications e.g., electronic devices, heat insulation, catalysis, waste management, sensor application, and so on [40]. 3D GO and rGO-based materials i.e., graphene aerogels (GAs) can integrate superior properties of graphene materials in continuous aerogel networks preventing aggregation and collapsing of graphene sheets. GAs are also sought in numerous applications including chemiluminescence detection of macromolecules [41], catalysis [42], electrode materials, energy storage, supercapacitor applications [43], and remediation of pollutants [44,45] such as dyes, pesticides, herbicides, and pathogens. Composites of GAs can be prepared by which the desired properties of guest molecules along with GAs can be combined in a single physical entity. Various nanoarchitectures and polymers were generated in situ within GAs such as CoFe_2_O_4_ nanoclusters [46], TiO_2_ [47], and Co [48] nanocrystals, gold nanoparticles [49], Fe_3_O_4_ nanoparticles [50], and quantum dots [51], along with some polymers such as polydimethylsiloxane [52], poly(aniline), poly(pyrrole) [34,53], and so on.

In situ synthesis of conductive polymers within 3D templating networks is of significant importance as the low stability of such polymers in freely standing forms was surmounted by the supporting matrix [54]. Besides the extra protection and stability incorporated within the composites, such kinds of hybrid structures integrate the inherent functionalities of their constituents into unique interfaces and exhibit superior properties than their precursors. Synthesis of various conductive polymers within porous networks has been reported such as poly(aniline), poly(pyrrole), and poly(thiophene) [55,56]. In the present study, the exceptional conductivity of p(p-PDA) was fortified by the extraordinary properties of GAs (high surface area and high porosity, robust and flexible mechanical properties, and the extremely light weight of the aerogel structure, and so on [57,58]).

Studies reported for CO_2_ detection in the literature are generally based on nondispersive infrared [59], electrochemical [60], optic [61], capacitive [62], and work function-based [63] sensors. Additionally, chemical sensors have received great attention in the detection of CO_2_. They give perceptible signals through gas-sensitive layers as changes in their conductivity, by which the amount of decrease or increase in their resistance or changes in their capacitive properties with the change of dielectric constants can be tracked upon CO_2_ gas exposure [64,65]. Because of the features such as low energy consumption, low cost, and flexible and adaptable size, which can be used in electronic applications, chemical sensors can afford noteworthy potentials for CO_2_ detection in different environments [66].

In this study, GOs were synthesized from graphite flakes by the modified Hummer method and then used in the fabrication of 3D highly porous GAs upon chemical reduction of the prepared GOs by L-Ascorbic acid (L-AA) as reducing agent and freeze-drying processes. Thereafter, a conductive polymer poly(para-phenylenediamine) (p(p-PDA)) was in situ synthesized within the pores of the prepared GAs followed by doping with several acids such as hydrochloric acid (HCl), nitric acid (HNO_3_), sulfuric acid (H_2_SO_4_), and phosphoric acid (H_3_PO_4_). The prepared GA/p(p-PDA) composites were characterized by Fourier transform infrared (FT-IR) spectroscopy, and thermal gravimetric (TG) analysis. The influences of different acid dopants on the electrical conductivity of GA/p(p-PDA) composites were determined and their sensory potentials against CO_2_ gas were investigated at ambient pressures and temperatures.

## 2. Materials and Methods

### 2.1. Materials

Graphite flakes (average particle size of 7–10 μm, 99% purity, Alfa Aesar), sodium nitrate (NaNO_3_, 99.0%–100.5%, Merck), sodium hydroxide (NaOH, 98%–100.5%, Sigma-Aldrich), potassium permanganate (KMnO_4_, 99%), hydrogen peroxide (H_2_O_2_, 30%, Merck), and sulfuric acid (H_2_SO_4_, 95%–97%, Sigma-Aldrich) were used in the preparation of GOs. L-Ascorbic acid (L-AA, 99%, Sigma Aldrich) was used for the reduction of GOs nanosheets in synthesis GAs. P-Phenylenediamine (p-PDA, 98%, Sigma Aldrich) was used as a monomer for in situ synthesis of p(p-PDA) in the GA networks, and ammonium persulfate (APS, 98%, Sigma Aldrich) was used as an initiator of oxidative p-PDA polymerization. Hydrochloric acid (HCl, 36%–38%, Sigma Aldrich), nitric acid (HNO_3_, 65%, Sigma Aldrich), sulfuric acid (H_2_SO_4_, 98%, Merck), and phosphoric acid (H_3_PO_4_, 85%, Sigma Aldrich) were used in the doping of in situ synthesized p(p-PDA) within GAs.

### 2.2. Synthesis of Graphene Aerogels

Graphene aerogels (GAs) were prepared by chemical reduction of prepared GOs following the cited literature with some modifications [34,67]. Concisely, 3.0 g of prepared GOs were dispersed in 500 mL DDW, following the modified Hummers method [39,68], with a final gravimetric concentration of 6 mg/mL. A quantity of 20 mL of this solution containing GO layers was transferred into graduated cylinders and subjected to sonication for 3 h at 30 °C–50 °C. After the sonication, the reducing agent L-AA was added into GO solution at a 3:1 w/w (L-AA:GO) ratio and sonicated for 1 min more to dissolve added L-AA. Then, the mixture was incubated without stirring at 40 °C for 24 h. Following the formation of black colored 3D graphene aerogel networks, they were transferred into 2.0 L beakers for washing in excess of DDW by continuously changing and replenishing the water every 8 h for 10 days. In the final step after washing, macroporous GAs were obtained via displacement of its liquid content with air by freeze-drying, and the resultant GAs were placed in closed tubes for further usage.

### 2.3. Synthesis of Conductive Poly(p-phenylenediamine) (p(p-PDA))

The synthesis of conductive poly(p-phenylenediamine) (p(p-PDA)) was carried out in accordance with the literature [69,70,71,72]. In short, 25 mL 0.5 M p(p-PDA) solutions were prepared in DDW. After that, 1 M 25 mL acid solutions containing 0.5 M APS were separately prepared from various acids such as HCl, HNO_3_, H_2_SO_4_, and H_3_PO_4_ and added drop by drop in separate p(p-PDA) solutions in an ice bath. Afterward, the prepared p(p-PDA) polymers were collected by centrifugation at 10,000 rpm for 10 min and washed twice with water and acetone, then dried by using a heat gun.

### 2.4. In Situ Synthesis of Conductive p(p-PDA) within GAs

For in situ synthesis of conductive p(p-PDA) polymers within GA networks, the earlier reports on p(p-PDA) synthesis were followed with some modifications [69,70,71,72]. Accordingly, the GAs were placed into 0.5 M 50 mL aqueous solutions of p-PDA and stirred for 1 h at 100 rpm in dark for loading of p-PDA monomers into pores and pore walls of the GAs. After that, the p-PDA loaded GAs were placed into 50 mL of different acid solutions containing 0.5 M APS and 1 M of various acids including HCl, HNO_3_, H_2_SO_4_, and H_3_PO_4_, then stirred for 30 min more at 100 rpm for in situ oxidative polymerization of p-PDA. Various acid-doped GA/p(p-PDA) composites were washed once with water and ethanol then dried in an oven at 50 °C.

### 2.5. Characterization

Morphological properties of GA-based conductive polymer composites were assessed by scanning electron microscopy (SEM, Hitachi, Regulus 8230) operating in a low vacuum mode at a 5 kV accelerating voltage. For the imaging, GA-based samples were mounted on aluminum SEM stubs with the aid of sticky carbon tapes, and the surface of the specimens was coated with gold to a few nanometers of thickness by a sputter coater to prevent excess charge buildup on the surface of specimens, and then samples were analyzed with SEM.

Functional groups of GAs and various acid-doped GA/p(p-PDA) composites were ascertained by FT-IR analysis and spectral peaks of these materials were recorded in the range of 650–4000 cm^−1^ with a 4 cm^−1^ resolution employing attenuated total reflectance of the attached FT-IR spectrometer (Thermo, Nicolet-iS10).

Thermal stabilities of GAs and various acid-doped GA/p(p-PDA) composites were determined by a TG analyzer (TGA, SII TG/DTA 6300, Exstar) under continuous purging of nitrogen gas with 200 mL/min flow and the heating rate of 10 °C/min up to 900 °C.

Electrical conductivities of GAs, and acid-doped GA/P(p-PDA) composites were measured at room temperature via a computer-controlled electrometer (Keithley 2400 Source-Meter).

### 2.6. Conductivity Measurements

Conductivity measurements of GAs and various acid-doped GA/p(p-PDA) composites with lengths of 1.24 ± 0.45 cm were performed by attaching small pieces of conductive carbon tape (~3 × 4 mm) to the top and bottom of bare and composite GA samples to connect the electrodes, followed by applying the voltage up to 40 V. The conductivity values were calculated from the slope of the current–voltage (I–V) response curves at the Ohmic (linear) region by using Equations (1) and (2);

V = I × R (1)

σ = (1/R) × (l/A) (2)

where, ‘V’ is the voltage, ‘I’ is the current, ‘R’ is the bulk resistance, ‘σ’ is the conductivity, ‘1/R’ is the resistivity, ‘l’ is the thickness and ‘A’ is the cross-sectional area of the sample.

### 2.7. Sensor Application of GA Based Composites to CO_2_ Gas

The potential sensor application of the prepared GA-based composites to CO_2_ gas was investigated by tracking the change in their conductivity upon CO_2_ exposure. For this purpose, 0.25 g of bare p(p-PDA) polymers were pelleted under compression of 10 tons of pressure, and the resulting pellet was exposed to CO_2_ gas for 30 min. The sensory potential of GA/p(p-PDA) composites were also evaluated by 30 min of CO_2_ gas exposure, which corresponded to 11,980 ppm CO_2_. The conductivity of p(p-PDA)s, and GA/p(p-PDA) composites, were determined by recording their current–voltage curves via an electrometer and subsequent calculation of conductivity values from Equations (1) and (2). The sensitivity studies of HCl-doped GA/p(p-PDA) composites were conducted by exposing them to CO_2_ gas for 0.5, 1, 5, 10, 20, 30, 45, and 60 min with the flow rate of 200 mL/min.

Besides, reusability of the GA/p(p-PDA)-HCl composites was also investigated. Briefly, the HCl-doped composite GA samples were treated with CO_2_ gas for 30 min, and the change in their conductivity was recorded. After that, the used (CO_2_ exposed) GA/p(p-PDA)-HCl composites were incubated at 60 °C for 2 h for regeneration of their conductivity and recovered conductivities were noted. Following the incubation, the composites were retreated with CO_2_ gas for another 30 min and the same reuse–regeneration cycles were applied for a total of 5 cycles.

## 3. Results and Discussion

### 3.1. In Situ Synthesis and Characterization of p(p-PDA) within GAs

**Figure 1:**
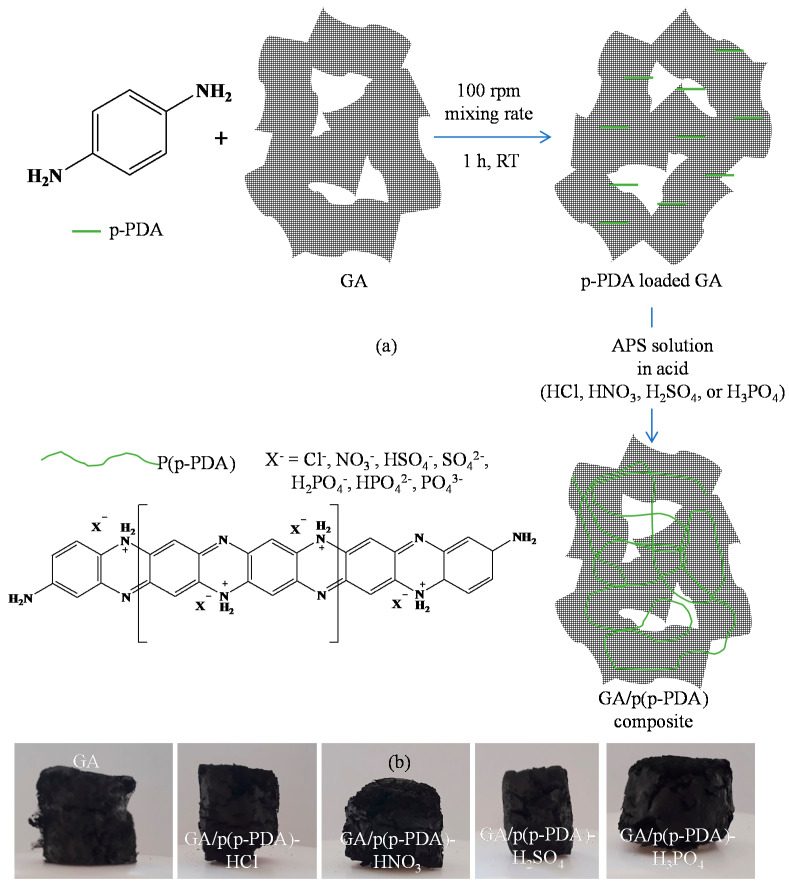
(**a**) Schematic illustration of GA/p(p-PDA) composite synthesis, and (**b**) digital camera images of GA/p(p-PDA) composites.

The schematic illustration describing the preparation of GA/p(p-PDA) composites was given in Figure 1. The loading of p-PDA monomer into 3D GA networks was governed by simple ionic interactions of partially positive p(p-PDA) monomers due to their amine groups with the partially negatively charged functional moieties of reduced GO sheets as well as hydrophobic interactions in GA networks, which was followed by oxidative polymerization in the presence of APS and various acid dopants such as HCl, HNO_3_, H_2_SO_4_, and H_3_PO_4_.

The amounts of in situ polymerized p(p-PDA) within GA pieces were gravimetrically calculated by the mass difference between dried bare GA and GA/p(p-PDA) composites and the results are reported in Table 1. As can be clearly noticed wherefrom, for 1.0 g of GA, the highest amount of p(p-PDA) polymers were synthesized in the presence of H_2_SO_4_ acid doping as 3.94 ± 0.8 g/g of GA.

In the descending order 3.67 ± 0.7 g, 2.08 ± 0.5 g, and 1.78 ± 0.3 g of p(p-PDA) polymers were in situ synthesized by simultaneous doping of H_3_PO_4_, HCl, and HNO_3_ acid solutions, respectively.

**Figure 2:**
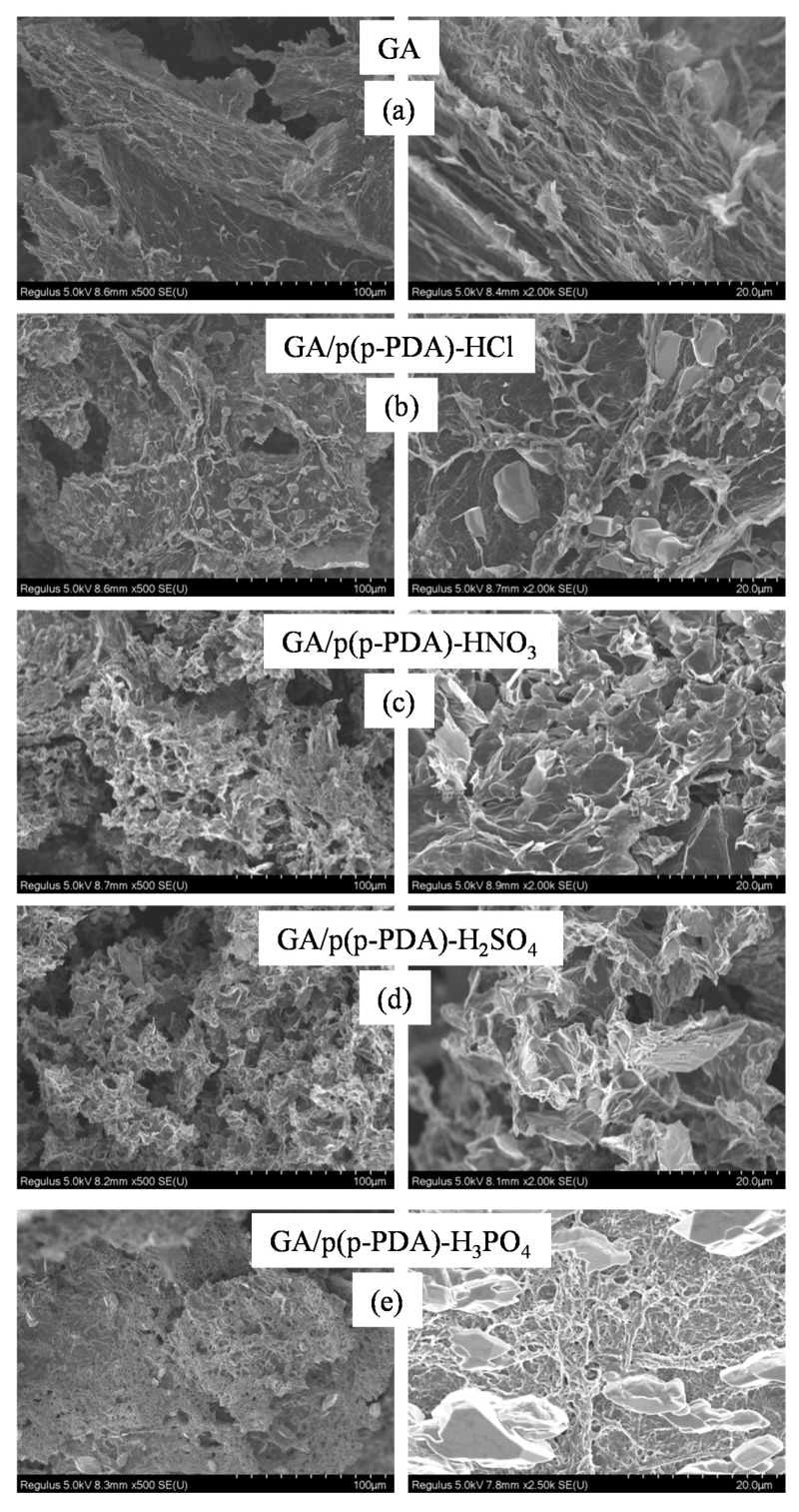
The SEM images of graphene aerogel (GA)-based composites; (**a**) GA, (**b**) GA/p(p-PDA)-HCl, (**c**) GA/p(p-PDA)-HNO_3_, (**d**) GA/p(p-PDA)-H_2_SO_4_, and (**e**) GA/p(p-PDA)-H_3_PO_4_.

Moreover, the SEM analysis of GA-based composites revealed the existence of highly porous structures with pores of varying sizes, in a range of 1–100 µm. The corresponding SEM images of GA, GA/p(p-PDA)-HCl, GA/p(p-PDA)-HNO_3_, GA/p(p-PDA)-H_2_SO_4_, and GA/p(p-PDA)-H_3_PO_4_ composites are given in Figure 2a–e, respectively. As shown in the micrographs, the pores and pore walls of the GA composites were covered with the in situ synthesized conductive p(p-PDA)s. The least amount of p(p-PDA) polymers appears to be the one that was synthesized in the presence of HNO3 acid doping with regards to other acid dopants.

It has been reported that high surface area, porosity, and pore volumes of GA-based materials favor diffusion of gases into the porous GA networks [73,74]. Thereby, these porous structures can be employed in various applications e.g., in situ synthesis of conductive polymers for potential sensor applications.

**Figure 3:**
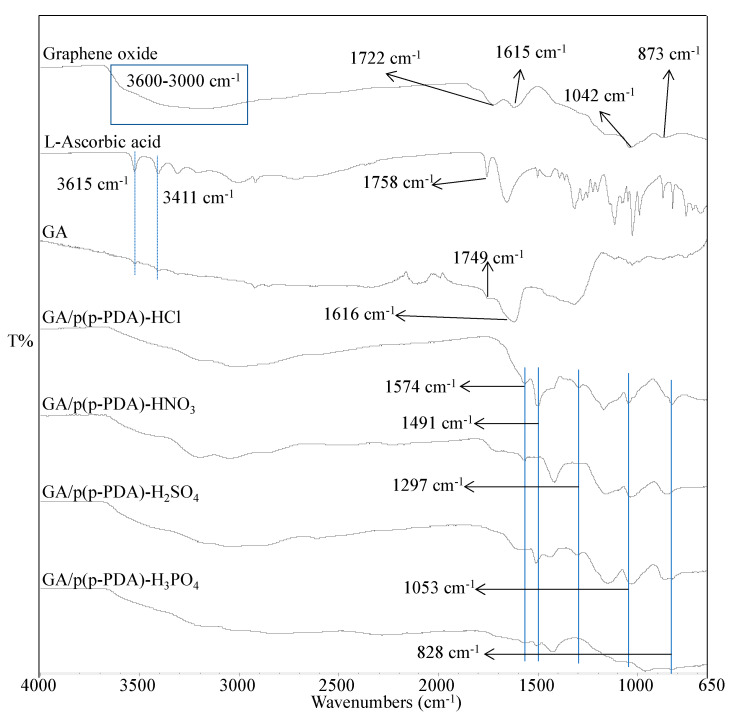
FT-IR spectra of GA/p(p-PDA) composites doped with various types of acids.

The FT-IR spectra of p(p-PDA) and prepared bare HCl-, HNO_3_-, H_2_SO_4_-, and H_3_PO_4_-doped conductive p(p-PDA) polymers are given in Figure S1. The characteristic peaks of p-PDA at 3389 cm^−1^ was assigned to symmetric stretching vibrations of =CH, and the peaks at 3032 cm^−1^ for symmetric and at 3302, and 3201 cm^−1^ were designated to asymmetric stretching vibrations of N-H bonds. The spectral peaks observed at 1613 cm^−1^ were attributed to N-H deformation of secondary amines, the peaks at 1502 cm^−1^ for symmetric stretching vibration of aromatic CC, the peaks at 1327 cm^−1^ for symmetric stretching of C-N, and the peaks that appeared at 807 cm^−1^ were assigned to out of plane deformation showing 1,4-distubistution in the benzene ring, respectively [75]. The FT-IR spectra of p(p-PDA) polymers doped with various types of acids have shown the same characteristic peaks as stretching deformations of quinone at 1589 cm^−1^, stretching vibrations of benzene at 1503 cm^−1^, and also the peak at 1303 cm^−1^ can be assigned to C-N stretching in a secondary aromatic amine, 1098 and 1014 cm^−1^ for the aromatic C-H in-plane bending modes, and 807 cm^−1^ for out-of-plane deformations of C-H in the 1,4-disubstituted benzene ring, respectively [76]. Moreover, the FT-IR spectrum of prepared GO, L-AA, GA, and GA/Pp(p-PDA) composites by using various types of acids as doping agents were compared in Figure 3.

As it is readily seen from the FT-IR spectrum of GO, the O-H stretching vibrations were observed as t broad bands between 3600–3000 cm^−1^, and the peak for C=O groups from both the carboxylic acid and ketones was observed at 1722 cm^−1^ [76,77]. The peak at 1615 cm^−1^ was assigned to the aromatic C=C bonds as a result of skeletal vibrations of unoxidized graphene units. The stretching vibrations at 1042 cm^−1^ and 873 cm^−1^, respectively, were attributed to C-O stretching modes of vibrations in the alkoxy and the ether groups of GO [77,78]. The FT-IR peaks that appeared at 3516, 3411, and 1645 cm^−1^ are assigned to O-H groups, and the peak observed at 1758 cm^−1^ can further stem from the C=O groups, which is noticeable from the FT-IR spectrum of L-AA [34]. For GA, the spectral peaks are seen at 3511 and 3408 cm^−1^ and are attributed to the O-H stretching, and the peaks at 1749 cm^−1^ belong to C=O stretching vibrations. The peaks that appeared at 1616 cm^−1^ can arise from the skeletal vibration of graphene sheets. From the oxidized graphene, GO sheets to their chemically reduced GA forms, the red shift can be readily noticed for C=O bands with the respective change from 1722 to 1749 cm^−1^ [34]. Additionally, the characteristic peaks of p(p-PDA), respectively, as stretching deformations of quinone at 1574 cm^−1^, stretching deformations of benzene at 1491 cm^−1^, and C-N stretching modes of a secondary aromatic amine at 1297 cm^−1^, aromatic C-H in-plane bending modes at 1053 cm^−1^, and the peaks at 828 cm^−1^ for out-of-plane deformations of C-H in the 1,4-disubstituted benzene ring can be realized from the given FT-IR spectra [76]. Based upon the systematical analyses of FT-IR spectra of the prepared GOs, GAs, and GA-p(p-PDA) composites, one can clearly state that the in situ syntheses of conductive p(p-PDA) polymers were successfully achieved within 3D GAs.

**Figure 4:**
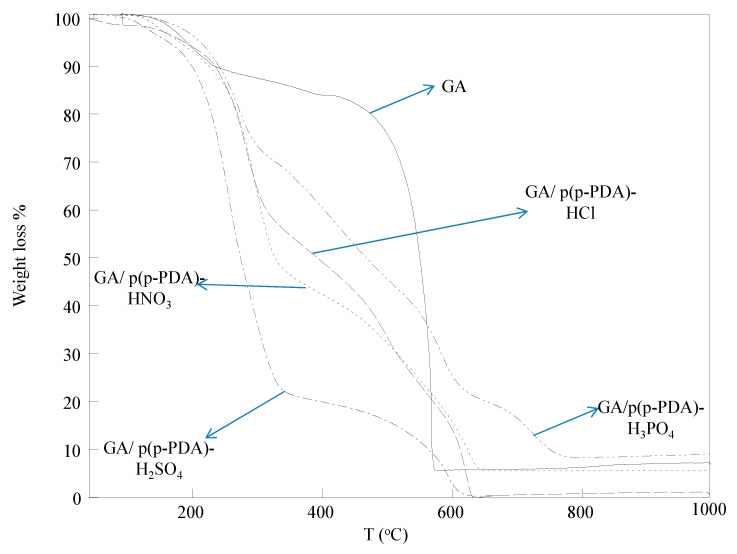
Thermal gravimetric (TG) thermograms of GA/p(p-PDA) composites doped with various types of acids.

The TGA thermograms of GA and various acid-doped GA/p(p-PDA) composites were compared in Figure 4. For GA, approximately 20% weight loss was observed at about 480 °C, and cumulatively 75% of its weight was lost up to heating 580 °C, and from thereon, no significant weight loss was observed by heating up to 1000 °C.

All of the GA/p(p-PDA) composites shared similar decomposition patterns. The first thermal decomposition of HCl-doped GA/p(p-PDA) composites was recorded between 30–225 °C with 8.4% weight loss, the second step was observed between 230–315 °C with 39.9% cumulative weight loss, and the last step was occurred between 320–630 °C with a final result of more than 99% cumulative weight loss. The HNO_3_-doped GA/p(p-PDA) composites were recorded to show 13.3% weight loss between 110–245 °C, the second and third steps of thermal decomposition occurred between 250–330 °C, and 340–640 °C with 50.1%, and 93.9% cumulative weight losses, respectively. H_2_SO_4_-doped GA/p(p-PDA) composites showed approximately 7.5% weight loss up to 185 °C, and heating up to 340 °C resulted in a cumulative weight loss of 77.4%. In addition, in the final decomposition step, more than 99% cumulative weight loss was observed by heating up to 630 °C. For the H_3_PO_4_-doped GA/p(p-PDA) composites, 24.3% weight loss was recorded between 130–290 °C. The second step occurred between 540–620 °C with 77.8% cumulative weight loss, and the last step of thermal decomposition was seen between 690–780 °C with a final cumulative weight loss of 91.5% (8.5% remaining residues). Moreover, according to TGA data obtained, H_2_SO_4_-doped GA/p(p-PDA) composites were the least thermally stable composite, which was followed by HCl-doped GA/p(p-PDA) composites. Comparatively, the H_3_PO_4_- and HNO_3_-doped GA/p(p-PDA) composites exhibited better thermal stability and higher remaining residues than the ones doped with H_2_SO_4_ and HCl, while all of the composites show similar patterns of thermal degradation. The differences seen within thermal degradation profiles of the GA/p(p-PDA) composites can be attributed to the distinctive effects of various acids dopants on the structure and morphology of the in situ synthesized conductive polymers. Particularly, as can be noticeable from the SEM images provided in Figure 2, the crystal structure of the GA-based polymer composites shows dopant-dependent variations, as it was also stated elsewhere that in situ synthesized conductive polymers can exhibit differences in their size and crystal structures with the effects of different doping acids [79].

### 3.2. Conductivity Measurements

**Figure 5:**
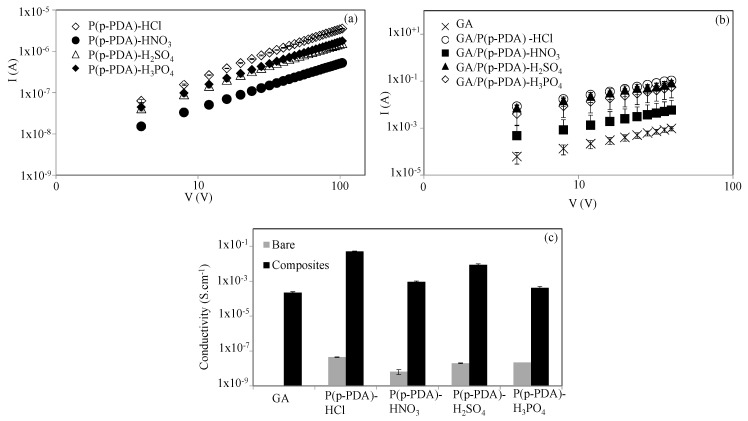
The current (I) versus voltage (V) plots of (**a**) p(p-PDA) polymers, (**b**) GA/p(p-PDA) composites and (**c**) comparison of calculated conductivity values for p(p-PDA) and GA/p(p-PDA) composites.

Carbon-based graphite, graphene oxide, graphene and/or graphene aerogels have three valence bands where electrons are in high attraction with the central nucleus. There is a free electron called π-electron that does not pass through the Fermi level as in the metals, however, the Fermi level has a narrow band that can easily shift the electron from the valance band to the conduction band and creates electron conductivity [80]. One of the most important properties of the conductivity of these carbon-based materials is that the in-plane electrical conductivity in their structure is much more than the out-of-plane electrical conductivity, mainly due to the low area between the graphitic layers, resulting from the weak π-electron bands connecting different layers of graphite [80,81]. On the other hand, in conductive polymers known as conjugated organic polymers, conductivity is associated with self-settling solitons, polarons, and bipolarons in the polymer chain [68,69]. These carriers become mobile in the electric field and move along the polymer chain, causing electrical conductivity. The I-V response plots of the various acid-doped p(p-PDA) and GA/p(p-PDA) composites are given in Figure 5a,b.

From these plots, the conductivity values for p(p-PDA) polymers and GA/p(p-PDA) composites the were calculated using Equations (1) and (2), respectively, and compared in Figure 5c. It was observed that the conductivity of GAs increased with the in situ synthesis of p(p-PDA). Electrical conductivities of bare p(p-PDA) and GA/p(p-PDA) composites were also given in Table 2. As given, the conductivities of p(p-PDA) polymers different from each other depending on the type of acid dopant used. The conductivities of p(p-PDA)-HCl, p(p-PDA)-HNO_3_, p(p-PDA)-H_2_SO_4_, and p(p-PDA)-H_3_PO_4_ were calculated as 4.46 × 10^−8^ ± 1.12 × 10^−8^, 6.46 × 10^−9^ ± 2.24 × 10^−9^, 1.99 × 10^−8^ ± 1.93 × 10^−9^, and 2.22 × 10^−8^ ± 1.11 × 10^−9^ S·cm^−1^, respectively. From these values, the highest conductivity was measured for the HCl-doped p(p-PDA) polymers, which is approximately 7-fold greater than HNO_3_, and 2-fold higher than the conductivities of H_2_SO_4_-, and H_3_PO_4_-doped p(p-PDA) polymers. Electrical conductivity of bare GAs was calculated as 2.17 × 10^−4^ ± 3.15 × 10^−5^ S·cm^−1^, which is much higher than all types of p(p-PDA) polymers with different acid doping. As was expected, conductivity of GAs underwent a significant rise after in situ preparation within GA of various acid-doped forms as GA/p(p-PDA) composites.

Conductivity of HCl-, HNO_3_- H_2_SO_4_-, and H_3_PO_4_-doped GA/p(p-PDA) composites were calculated to be 5.16 × 10^−2^ ± 2.72 × 10^−3^ S·cm^−1^, 9.19 × 10^−4^ ± 1.29 × 10^−4^ S·cm^−1^, 8.78 × 10^−3^ ± 1.17 × 10^−3^ S·cm^−1^, and 4.11 × 10^−4^ ± 9.13 × 10^−5^ S·cm^−1^, respectively. These values were approximately 250-, 4-, 40-, and 2-fold greater than the conductivity of bare GAs, respectively. As it is in a good agreement with the conductivity of HCl-doped bare p(p-PDA) polymers, the conductivity of in situ prepared HCl-doped GA/p(p-PDA) composites with 5.16 × 10^−2^ ± 2.72 × 10^−3^ S·cm^−1^ is approximately 60-fold, 6-fold, and 125-fold higher than those of HNO_3_-, H_2_SO_4_-, and H_3_PO_4_-doped GA/P(p-PDA) composites, respectively. Obviously, different types of acid dopants have imparted distinct conductivity values to the GA based composites. The higher conductivity was observed for in situ prepared HCl-doped GA/p(p-PDA) composites. As mentioned earlier, in the literature, the types of the used acids for doping of conductive polymers have significant effects on size and crystal structure of the conductive polymers, and higher crystallinity was observed in HCl-doped conductive polymers amongst doping of several others [79]. As it is clearly seen from Table 1 and SEM images of the GA-based composites, the amount of in situ prepared HCl-doped p(p-PDA) is more than the in situ prepared amounts within HNO_3_-doped p(p-PDA) GAs, so is its conductivity. Therewithal, the smaller size of HCl than other used acids can make it a better dopant as it can more readily diffuse into pores of GA composites. Therefore, the highest conductivity values were observed in in situ prepared HCl-doped GA/p(p-PDA) composites.

### 3.3. Conductivity Change of GA Based Composites in Response to CO_2_ Gas Exposure

**Figure 6:**
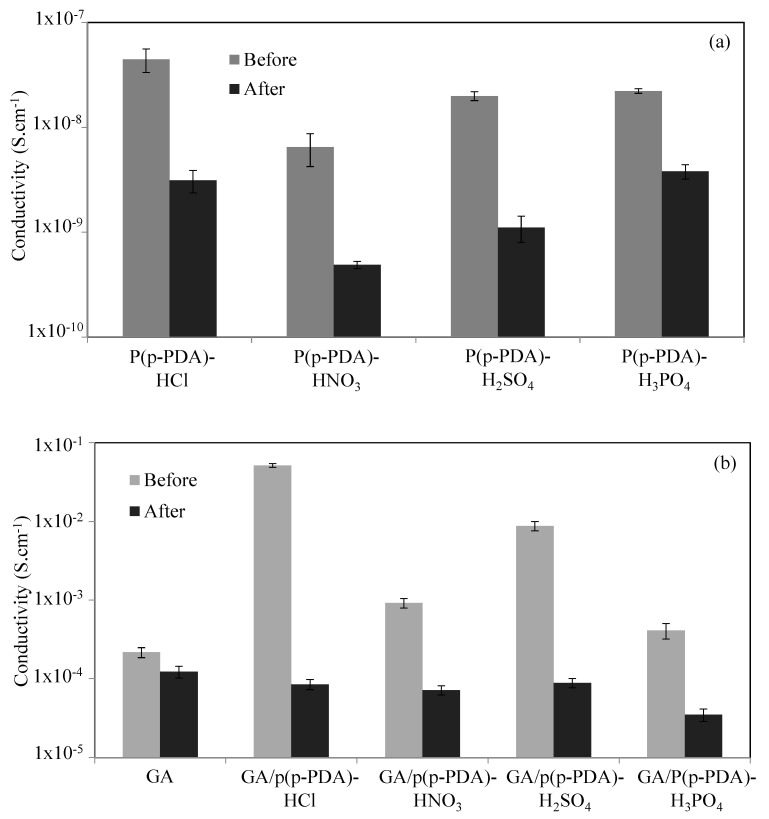
The conductivities of (**a**) p(p-PDA) polymers, and (**b**) GA/p(p-PDA) composites before and after 30 min of CO_2_ gas exposure.

Carbon dioxide (CO_2_) gas, as one of the most dangerous causatives of global warming, is increasing continuously in the atmosphere. According to the standards reported by the American Association of Heating, Refrigerating and Air Conditioning Engineers (ASHRAE), the concentration of CO_2_ required for healthy breathing should not exceed 1000 ppm [82]. However, according to the results of extensive research and measurements, the amount of industrial CO_2_ emission is many times higher than this threshold. The CO_2_ concentration in the atmosphere increased from 414 ppm in May 2019 to 417 ppm in May 2020 [83]. It is urgent to take serious precautions to cope with this steady CO_2_ increase in the atmosphere. Therefore, the design and use of functional materials that can sense, capture, and store the ways in which the emission of pollutant gases, whose concentration in the atmosphere intensifies daily, endangers the lives of all living creatures. Therefore, CO_2_ sensors and adsorbent potentials of the prepared bare p(p-PDA) polymers, GAs, and GA/p(p-PDA) composites were evaluated by exposing them to CO_2_ gas and the changes in their conductivity were examined. The experimental setup used in the CO_2_ exposure of prepared materials was reported in the literature [84]. The HCl-, HNO_3_-, H2SO_4_-, and H_3_PO_4_-doped p(p-PDA) polymers and their GA composites of known conductivities were exposed to CO_2_ gas for 30 min at 100 mL/min flow rate and the variations in their conductivities in response to CO_2_ gas exposure were illustrated in Figure 6a,b, respectively. It was clearly seen that the conductivity of various acid-doped P(p-PDA) polymers and GA/P(p-PDA) composites decreased after 30 min of CO_2_ exposure. The extent of conductivity falls of these acid-doped P(p-PDA) polymers and GA/P(p-PDA) composites are given in Table 2. As it was realized therefrom, an approximately 15-fold decrease in the conductivity of HCl-doped P(p-PDA) was observed from 4.46 × 10^−8^ ± 1.12 × 10^−8^ S·cm^−1^ to 3.12 × 10^−9^ ± 7.57 × 10^−10^ S·cm^−1^ after 30 min of CO_2_ gas exposure. Similarly, HNO_3_- and H_2_SO_4_-doped P(p-PDA) polymers have experienced, respectively, about 13-, and 18-fold decrease after 30 min of CO_2_ gas exposure. On the other hand, the decrease in conductivity of H_3_PO_4_-doped P(p-PDA) was slightly lower than that of the others with approximately 6-fold reduction.

Moreover, it was found that the conductivity of bare GA was calculated to be 2.17 × 10^−4^ ± 3.15 × 10^−5^, and 1.23 × 10^−4^ ± 2.11 × 10^−5^ S·cm^−1^, respectively, before and after 30 min CO_2_ gas exposure, indicating poor sensitivity of bare GA to CO_2_ gas. On the other hand, approximately 600-fold decrease in the conductivity of HCl-doped GA/p(p-PDA) composites from 5.16 × 10^−2^ ± 2.72 × 10^−3^ S·cm^−1^ to 8.52 × 10^−5^ ± 1.21 × 10^−5^ S·cm^−1^ after 30 min of CO_2_ gas exposure were attained.

Additionally, the conductivity of H_2_SO_4_-doped GA/p(p-PDA) composites appeared to show an approximately 100-fold decrease from 8.78 × 10^−3^ ± 1.17 × 10^−3^ to 8.91 × 10^−5^ ± 1.19 × 10^−5^ S·cm^−1^ after 30 min of CO_2_ gas exposure, whereas the changes in the conductivity of HNO_3_-, and H_3_PO_4_-doped GA/P(p-PDA) composites were far less than the others, with a 13- and 12-fold decrease, respectively, after 30 min exposure to CO_2_ gas.

The change in the conductivity of GA/p(p-PDA) composites in the course of CO_2_ exposure can be explained by diffusion of the gas within aerogel networks, which were the main reason that more conductivity reduction has been observed in GA/p(p-PDA) composites as compared to those of the free-standing p(p-PDA) polymers. More specifically, the CO_2_ gas could not diffuse into the interiors of the bare p(p-PDA) pellets, yet as a result of much higher surface area and macroporous structure of GA/p(p-PDA) composites along with the gas adsorption properties of the graphite and graphene oxide layers. Consequently, larger amounts of the CO_2_ gas were able to diffuse towards the porous network of the HCl- and H_2_SO_4_-doped GA/p(p-PDA) composites and taped the pores much better. In addition, the positive charges, and unpaired electrons of in situ synthesized p(p-PDA) within GAs can easily interact with CO_2_ molecules, which have π-type C=O binding ability and a lone pair of electrons [85].

**Figure 7:**
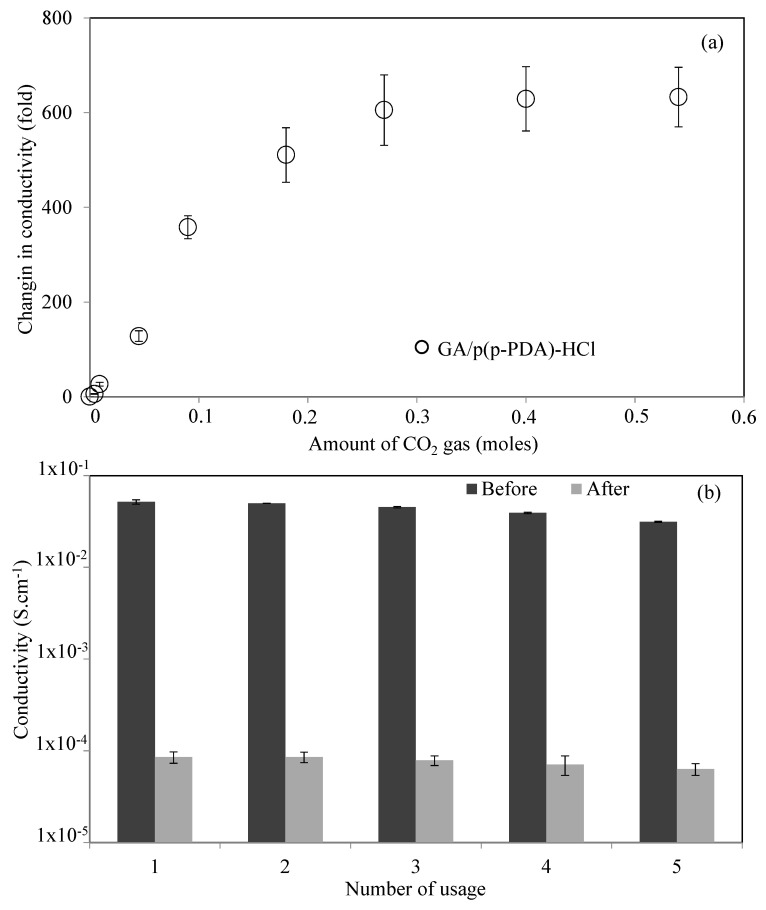
(**a**) The change in the conductivity of GA/p(p-PDA)-HCl composites in the presence of the various amount of CO_2_ gas, and (**b**) reusability of GA/p(p-PDA)-HCl composites.

The response of GA-based composites can be defined as a change in the conductivity in the presence of various amounts of CO_2_ molecules, and sensitivity can be defined as the ratio between the output signal (conductivity) per mole of the sensed molecule, CO_2_. Hence, the HCl-doped GA/p(p-PDA) composites exhibiting the greatest change in conductivity was studied for their sensitivity towards CO_2_ gas, and the change in the conductivity of GA/p(p-PDA)-HCl composites was recorded at different exposure time scales, 0.5, 1, 5, 10, 20, 30, 45, 60 min at 200 mL/min CO_2_ flow rate, and the corresponding CO_2_ sensitivity graph is presented in Figure 7a. The amounts of CO_2_ exposed to GA/p(p-PDA)-HCl composites at 0.5, 1, 5, 10, 20, 30, 45 and 60 min periods were calculated as 0.0045, 0.009, 0.045, 0.09, 0.18, 0.27, 0.40, and 0.54 moles, respectively. It was observed that the change in conductivity of GA/p(p-PDA)-HCl composites were increased with the increase in the time of CO_2_ gas exposure. Accordingly, 5.7-, 26.4-, 128-, 358-, 511-, 605-, 629-, and 633-fold conductivity decreases were recorded for GA/p(p-PDA)-HCl composites upon exposure to 198, 396, 1980, 3960, 7920, 11,980, 17,820, and 23,960 ppm CO_2_, respectively. The required concentration of CO_2_ for a healthy breath was stated to be less than 1000 ppm, and the concentration of CO_2_ in the atmosphere was 417 ppm as of May 2020. According to the sensitivity graph of HCl-doped GA/p(p-PDA) composites, 193.6 ppm is enough to trigger a 5.7-fold decrease in the conductivity of GA/p(p-PDA)-HCl composites and gradually decreased e.g., a 26.4- and 128- fold decrease can be monitored at 397.2, and 1936 ppm CO_2_ concentration in only 1 and 5 min exposures, respectively. These results clearly indicate that GA/p(p-PDA)-HCl composites can be used as a promising candidate for CO_2_ detection in various environmental applications including the detection of CO_2_ in the atmosphere. In here, the GA/p(p-PDA)-HCl as CO_2_ sensor is based on the change in conductivity upon CO_2_ exposure in a concentration-dependent manner, so that the sensitivity can be calculated as conductivity changes (fold)mole of CO_2_. The sensitivity of GA/p(p-PDA)-HCl is calculated from the slope of Figure 7a, and it was observed that there was a 1288.7-fold of conductivity change(decrease)/mole of CO_2_ gas.

Furthermore, the reusability of the prepared GA/p(p-PDA)-HCl composites were investigated for five successive (re)use and regeneration cycles, and the corresponding graph is illustrated in Figure 7b. It was observed that the conductivity of GA/p(p-PDA)-HCl composites decreased from 5.16 × 10^−2^ ± 2.72 × 10^−3^ S·cm^−1^ to 8.52 × 10^−5^ ± 1.21 × 10^−5^ S·cm^−1^ after 30 min of CO_2_ gas exposure, and was recovered to 4.97 × 10^−2^ ± 1.13 × 10^−3^ S·cm^−1^ after 2 h incubation at 60 °C. The conductivity of GA/p(p-PDA)-HCl composites decreased from 4.97 × 10^−2^ ± 1.13 × 10^−3^ S·cm^−1^ to 8.54 × 10^−5^ ± 1.11 × 10^−5^ S·cm^−1^ after the 2nd cycle of CO_2_ gas exposure, and was recovered back to 4.51 × 10^−2^ ± 9.35 × 10^−4^ S·cm^−1^ after the 2nd cycle. Then, the conductivity of the composites decreased to 7.87 × 10^−5^ ± 9.84 × 10^−6^ S·cm^−1^ after the 3rd CO_2_ gas exposure, and it was again increased to 3.91 × 10^−2^ ± 8.11 × 10^−4^ S·cm^−1^ after the 3rd regeneration. The conductivity of GA/p(p-PDA)-HCl composites decreased from 3.91 × 10^−2^ ± 8.11 × 10^−4^ S·cm^−1^ to 7.11 × 10^−5^ ± 1.71 × 10^−5^ S·cm^−1^ in the 4th cycle of CO_2_ exposure, and then restored to 3.13 × 10^−2^ ± 4.01 × 10^−4^ S·cm^−1^ after the 4th recovery. Finally, the conductivity decreased to 6.32 × 10^−5^ ± 9.23 × 10^−6^ S·cm^−1^ after the 5th 30 min of CO_2_ gas exposure. These findings clearly demonstrated that the GA/p(p-PDA)-HCl composites can be used in the detection of CO_2_ gas repeatedly after a 2 h of incubation at 60 °C, with almost full recovery to its initial conductivity measuring capability.

## 4. Conclusions

Herein, we demonstrated the potential usage of GAs as templates and sensory materials. Using GAs as templates in the in situ synthesis of conductive p(p-PDA) polymers clearly reveals the the versatility of these materials. The acid types, HCl, HNO_3_, H_2_SO_4_, and H_3_PO_4_ used in doping of p(p-PDA) polymers were found to have a considerable influence on the electrical conductivity of both p(p-PDA) polymers and their corresponding GA-based composites. GA/p(p-PDA) composites achieved higher electrical conductivities than both their bare p(p-PDA) polymers and bare GAs. The in situ synthesis of GA/p(p-PDA)-HCl composites gave rise to a 250-fold increase in the conductivity of bare GAs from 2.17 × 10^−4^ ± 3.15 × 10^−5^ S·cm^−1^ to 5.16 × 10^−2^ ± 2.72 × 10^−3^ S·cm^−1^. Moreover, GA/P(p-PDA)-HCl composites were revealed to show the best response to CO_2_ gas amongst other acid-doped composites, with an approximately 600-fold conductivity decrease. Owing to the well-known properties of graphene oxides and GAs to absorb gases, and the response of in situ prepared p(p-PDA) conductive polymer, GA/p(p-PDA) composites were shown to be a sensor for CO_2_ gas with the capability of measuring CO_2_ levels in the atmosphere, which are well within its sensitivity level. Although the gravimetric amount of in situ synthesized p(p-PDA) polymers with HCl doping in GA/p(p-PDA) composites is less than that of H_2_SO_4_- and H_3_PO_4_-doped GA/p(p-PDA) composites, better electrical conductivity, as well as better CO_2_ response of this material, affords better outcomes in terms of real applications. Furthermore, GA/p(p-PDA)-HCl composites exhibited a 5.7-fold conductivity decrease even at 193.6 ppm CO_2_ gas concentration in only 30 sec, and a 26.4-fold decrease in 1 min at 397.2 ppm CO_2_ concentration, along with the sensitivity of 1288.7 fold conductivity decrease/mole of CO_2_ gas. Since these concentrations are less than the half of those reported as dangerous, GA/p(p-PDA)-HCl composites hold remarkable potential in the determination CO_2_ as a sensor in many industrial effluents and mining applications, and so on.

Overall, it is apparent that GA-based materials offer a lot of potential, as they are not restricted to CO_2_ sensors and detectors in chimneys of factories, dwellings, and lab gadgets such as CO_2_ incubators, etc., to determine the level of emitted/generated or released CO_2_, but they may also be useful to track accumulated levels of CO_2_ in different milieus, including climate chambers and indoor living environments, and so on.

## Figures and Tables

**Table 1:** The gravimetric amounts of in situ synthesized p(p-PDA) polymers within GAs.

Type of Dopant	GA (g)	GA/p(p-PDA) (g)	In Situ Synthesized p(p-PDA) (g/g)
HCl	0.04 ± 0.002	0.12 ± 0.04	2.08 ± 0.5
HNO_3_	0.03 ± 0.002	0.08 ± 0.01	1.78 ± 0.3
H_2_SO_4_	0.03 ± 0.001	0.15 ± 0.03	3.94 ± 0.8
H_3_PO_4_	0.03 ± 0.002	0.14 ± 0.04	3.67 ± 0.7

**Table 2:** The change in conductivities of p(p-PDA) polymers, and GA/p(p-PDA) composites upon 30 min of CO_2_ exposure.

Type of Dopant	Conductivity (S·cm^−1^)		Decrease in Conductivity (fold)
	P(p-PDA)		
	Before	After	
HCl	4.46 × 10^−8^ ± 1.12 × 10^−8^	3.12 × 10^−9^ ± 7.54 × 10^−10^	~14
HNO_3_	6.46 × 10^−9^ ± 2.24 × 10^−9^	4.89 × 10^−10^ ± 3.89 × 10^−11^	~13
H_2_SO_4_	1.99 × 10^−8^ ± 1.93 × 10^−9^	1.11 × 10^−9^ ± 3.11 × 10^−10^	~18
H_3_PO_4_	2.22 × 10^−8^ ± 1.11 × 10^−9^	3.81 × 10^−9^ ± 5.99 × 10^−10^	~6
Materials	GA based composites		Decrease in conductivity (fold)
	Before	After	
GA	2.17 × 10^−4^ ± 3.15 × 10^−5^	1.23 × 10^−4^ ± 2.11 × 10^−5^	~2
GA/p(pPDA)-HCl	5.16 × 10^−2^ ± 2.72 × 10^−3^	8.52 × 10^−5^ ± 1.21 × 10^−5^	~600
GA/p(pPDA)-HNO_3_	9.19 × 10^−4^ ± 1.29 × 10^−4^	7.23 × 10^−5^ ± 9.88 × 10^−6^	~13
GA/p(pPDA)-H_2_SO_4_	8.78 × 10^−3^ ± 1.17 × 10^−3^	8.91 × 10^−5^ ± 1.19 × 10^−5^	~100
GA/p(pPDA)-H_3_PO_4_	4.11 × 10^−4^ ± 9.13 × 10^−5^	3.51 × 10^−5^ ± 6.33 × 10^−6^	~12

## Supplementary Materials

The following are available online at https://www.mdpi.com/2072-666X/11/7/626/s1, Figure S1: The FT-IR spectrum of bare p(p-PDA) polymers doped with various types of acids.
