# Supplementary figures and images for: Graphene Aerogels for In Situ Synthesis of Conductive Poly(para-phenylenediamine) Polymers, and Their Sensor Application
Source: Micromachines (Basel). 2020 Jun 27;11(7):626. doi: 10.3390/mi11070626 (PMC7408166; doi:10.3390/mi11070626)

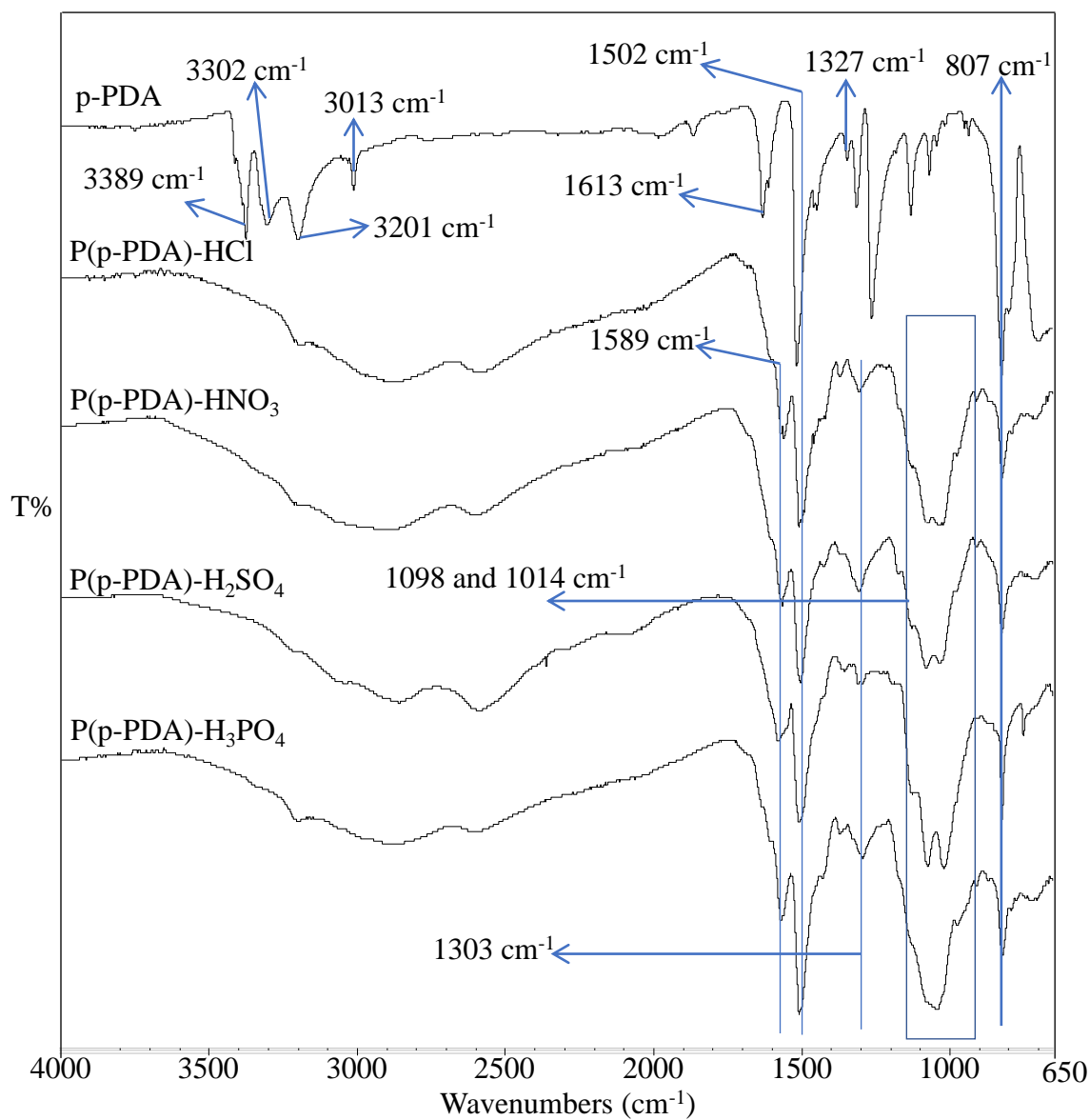

Figure S1: The FT-IR spectra of p(p-PDA) polymers doped with various types of acids.

Supplement: Supplementary file 1 [file micromachines-11-00626-s001.pdf]
